# Identifying Novel Estrogenic Mitochondrial Targets in Hypothalamic Proopiomelanocortin Neurons by Chemoproteomics

**DOI:** 10.64898/2026.06.17.732817

**Published:** 2026-08-21

**Authors:** Jian Qiu, Martha A Bosch, Mya S Wolfe, Ksenija Korac, Stefano Rizzo, Todd L. Stincic, Scotland E. Farley, Wendy Fitzgerald, Megha Rajendran, Aurelien Laguerre, Frank Stein, Philip F. Copenhaver, Oline K Ronnekleiv, Sean M Ronnekleiv-Kelly, Tatiana Rostovtseva, Sergey Bezrukov, Carsten Schultz, Martin J Kelly

## Abstract

Loss of estrogens at menopause is linked to impaired brain metabolism and increased risk of Alzheimer’s disease (AD). However, estrogen replacement therapies are limited due to the deleterious effects of estrogen on peripheral organs and increased risk of vascular dementia. We have developed a non-steroidal estrogenic compound, STX, which does not bind to the classical estrogen receptors α and β, but mimics estrogenic signaling in the central nervous system (CNS) without the peripheral reproductive actions. STX is protective against neurodegeneration in stroke and AD models, but its molecular targets are unknown. Here, we identified and validated STX neural targets using chemoproteomic, molecular biological, electrophysiological and metabolic assays of hypothalamic proopiomelanocortin (POMC) neurons. Chemoproteomic profiling identified voltage dependent anion channels (VDAC1-3) as major intracellular binding partners in mHypo43 (POMC) cells. Based on quantitative single-cell PCR, Vdac2 was identified as the dominant isoform in female hypothalamic POMC neurons. Seahorse metabolic flux analyses showed that STX potently increased glycolysis, oxidative respiration and mitochondrial ATP production in mHypo43 cells. Nanomolar concentrations of STX enhanced VDAC2 voltage-dependent gating in reconstituted lipid membranes and shifted the low-conductance states toward anion selectivity, consistent with increased ATP flux. Together, these findings reveal a mechanism for the neuroprotective effects of STX through enhancing mitochondrial bioenergetics and modulating VDAC channel properties, potentially increasing cellular energy stores. Therefore, this work identifies previously unrecognized estrogenic mitochondrial targets and provides a mechanistic basis for the neuroprotective actions of STX relevant to menopause-associated brain vulnerability.

## Full Text Availability

The license terms selected by the author(s) for this preprint version do not permit archiving in PMC. The full text is available from the preprint server.

